# Obesity and COVID-19 mortality are correlated

**DOI:** 10.1038/s41598-023-33093-3

**Published:** 2023-04-11

**Authors:** Bernard Arulanandam, Hamid Beladi, Avik Chakrabarti

**Affiliations:** 1 Tufts University School of Medicine, Boston, MA USA; 2 University of Texas at San Antonio, San Antonio, USA; 3 University of Wisconsin-Milwaukee (UWM), University of Wisconsin, 836 Bolton Hall, Milwaukee, WI 53201 USA; 4 Affiliated Faculty, Northwestern Mutual Data Science Institute, UWM, Milwaukee, USA

**Keywords:** Diseases, Infectious diseases, Viral infection

## Abstract

In view of a conspicuous absence of any cross-country study linking obesity and COVID-19 mortality, we conduct an empirical analysis of plausible associations between COVID-19 mortality and the proportion of obese in the adult population distributed across 142 countries around the globe. We observe a statistically significant positive association between COVID-19 mortality and the proportion of obese in adult populations spanning 142 countries. This association holds across countries belonging to different income groups and is not sensitive to a population’s median age, proportion of the elderly, and/or proportion of females. The estimated elasticity of COVID-19 mortality, with respect to the proportion of obese in adult populations, is the highest for the sub-sample of countries that belong to the high-income group. While limits of confidence intervals around the point estimates of these elasticities range between 0.7 and 2.1, on an average, every percentage point increment in the proportion of obese in adult populations contributes to an additional 1.5% points to COVID-19 mortality for high income countries. A positive association, observed between COVID-19 mortality and the proportion of the obese in a country’s adult population, is robust subject to alterations in the conditioning information set on age, gender, and income.

## Introduction

A close look at this vast and growing body of literature on severe acute respiratory syndrome coronavirus 2 (COVID-19) reveals the sparsity of empirical studies on links between obesity and COVID-19 mortality across countries. With more than 6.5 million lives lost during the current pandemic, in this paper, we turn the spotlight on obesity as a determinant of cross-country variations in mortality due to COVID-19. The relevant medical literature provides a point of reference for us to conduct the first cross-country study linking mortality due to COVID-19 and the percentage of the adult population that are obese.

**Figure 1:**
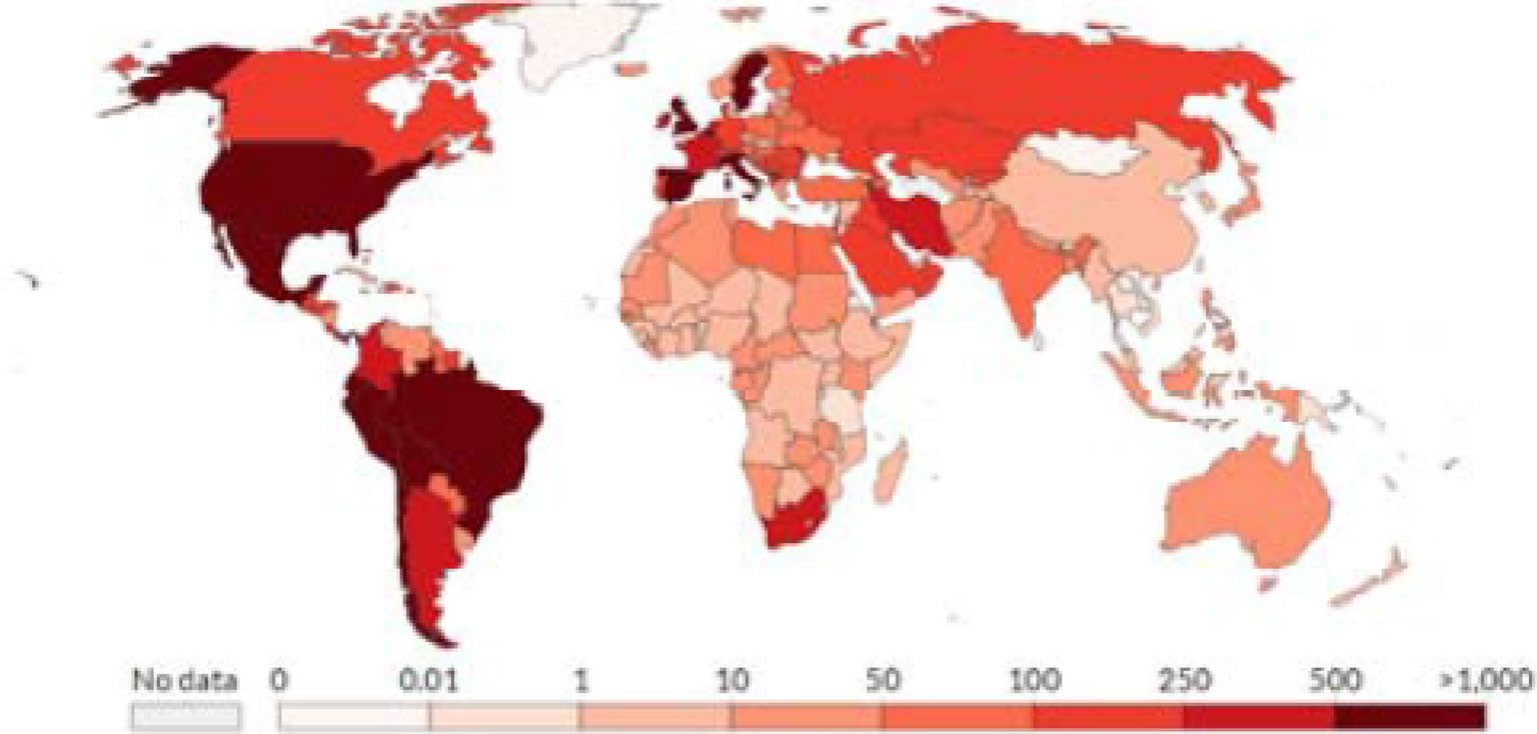
Global Distribution of COVID-19 Deaths Per Million People. Source: European Centre for Disease Prevention and Control.

**Figure 2:**
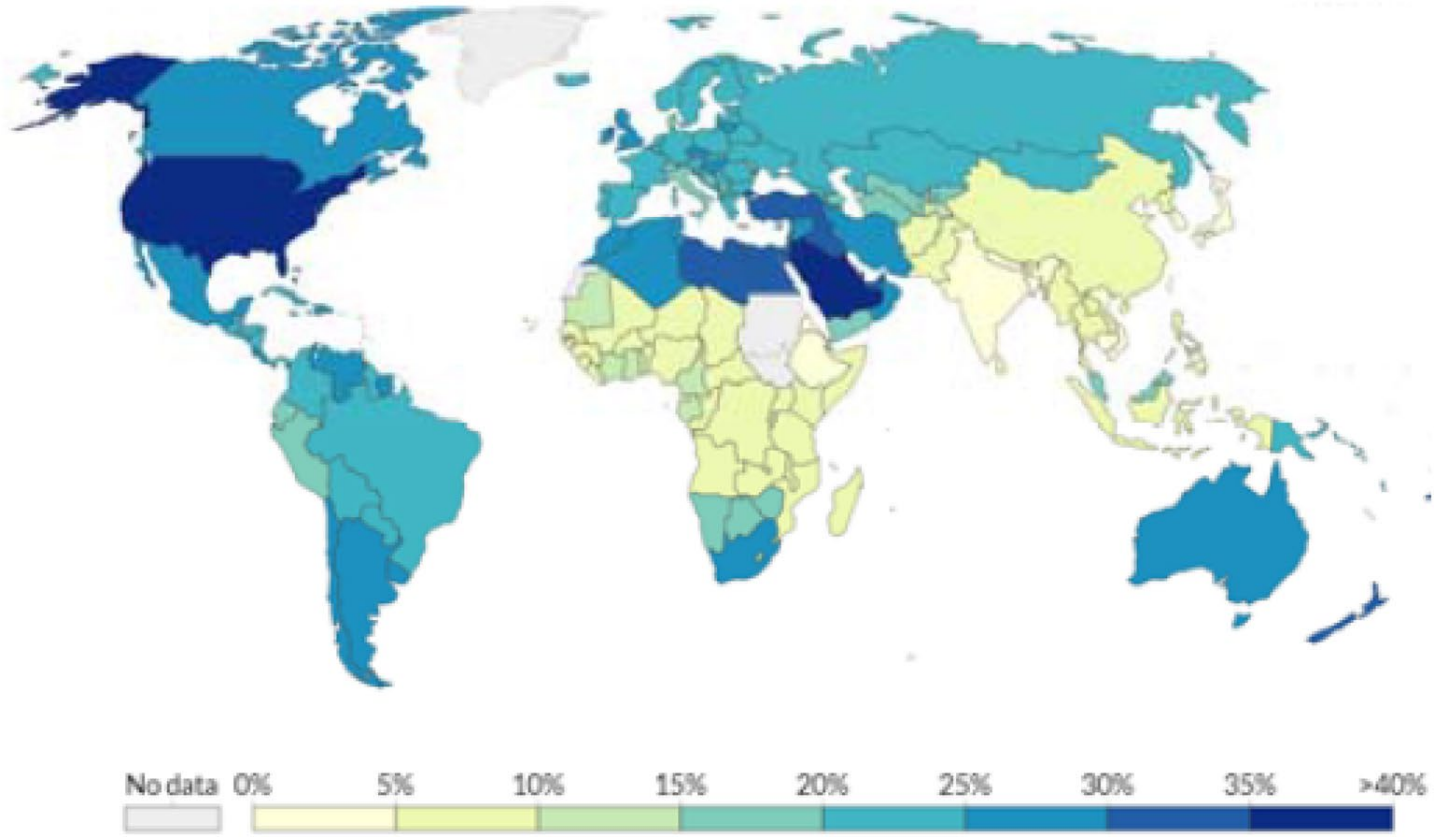
Global Distribution of Obese in Adult Population. Source: Global Health Observatory, World Health Organization.

Obesity is a major public health concern: at least 11% of men and 15% of women, more than half a billion adults, are obese worldwide. Lockhart and O’Rahilly^1^ point out that, although emerging evidence suggests that the obese are at heightened risk of dying from COVID-19, “the mechanisms underlying this are poorly understood.” Figures 1 and 2 (mapping the spatial distribution of COVID-19 mortality and that of obese adult population, across countries, respectively) reflect a remarkable resemblance: obese adults are concentrated mostly in relatively rich countries while lower income countries are home to leaner adults; COVID-19 mortality is typically higher in richer countries and lower in lower income countries. Nearly two-thirds of adults are obese in most high-income countries while low-income and lower-middle income countries, that constitute nearly half of the global population, have accounted for only two percent of the global death toll attributed to COVID-19.

**Figure 3:**
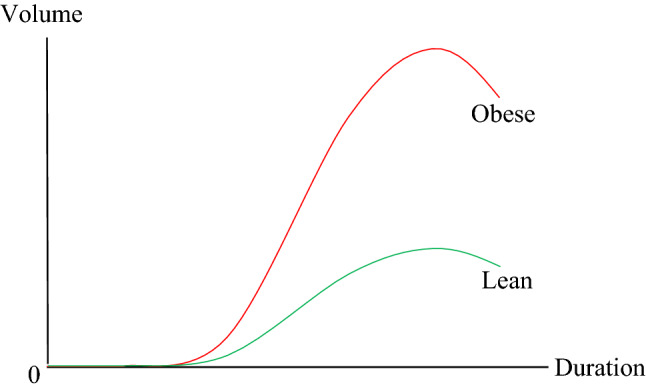
Volume and Duration of Viral Shedding: Obese vs. Lean^6^. Source: Honce and Stacey.

Anecdotal evidence aside, in view of the sparsity of formal analyses of correlations between the percentage of obese in adult populations and mortality due to COVID-19 across nations, our contribution can be linked to the relevant medical studies that have looked into clinical mechanisms that can explain the role of obesity in mortality due to COVID-19. From a clinical standpoint, obesity can be linked to multiple comorbidities that can result in COVID-19 related death. Since obesity may be responsible for a higher volume and extended duration of viral shedding, as shown in Fig. 3, it can result in a greater exposure as well. Bhatraju et al.^2^, Caussy et al.^3^, Chen et al.^4^ and Muscogiuri G et al.^5^ provide convincing evidence suggesting that outcomes with COVID-19 are worse for the overweight while hospital reports have been indicative of the fact that the likelihood of survival is relatively low among obese COVID-19 patients.

With this backdrop, to the best of our knowledge, we engage in the first cross-country study of correlations between the percentage of adult population that is obese and mortality due to COVID-19. In comparison, there is an expanding pool of studies that use individual-level data in the estimation of associations between prevalence of obesity and COVID-19 mortality. However, the literature is controversial and meta-analyses^7–10^ of these studies have yielded conflicting conclusions. In the following section, we describe the data and empirical analysis. We collect our findings in Section “Results”. The penultimate section underscores the importance of revisiting public policy with due cognizance of public health crises, in the light of our findings on associations between COVID-19 mortality and obesity. In the final section, we conclude.

## Data and empirics

All data used, in this paper, are publicly available. Observations on mortality due to COVID-19, defined in terms of deaths (per million people) due to COVID-19, is extracted from the European Centre for Disease Prevention and Control (ECDC). Data on the percentage of obese adults in populations, drawn from estimates of the prevalence of obese women and men among those 18 years and older, is obtained from The Global Health Observatory (GHO) repository. An individual is identified as being obese his/her the body mass index (BMI) is at least 30 kg/m^2^, where BMI is clinically defined as person’s weight in kilograms divided by his or her height in meters squared. Population characteristics (i.e. median age, percentage of adults aged at least 65 years, and proportion of females) are based on estimates provided by the World Population Prospects, of the United Nations Population Division. Country income groups (high income, upper middle income, lower middle income, and low income) are based on 2019 per capita Gross National Income (GNI), following the Atlas method of classification adopted by World Bank. Countries with a per capita GNI of $12,536 or more are identified as high income; those with a per capita GNI ranging from $4046 to $12,535 belong to the upper middle-income category; those with a per capita GNI ranging from $1036 to $4045 are labelled as lower middle-income countries; and those with a per capita GNI of $1035 or less constitute low-income countries.

**Figure 4:**
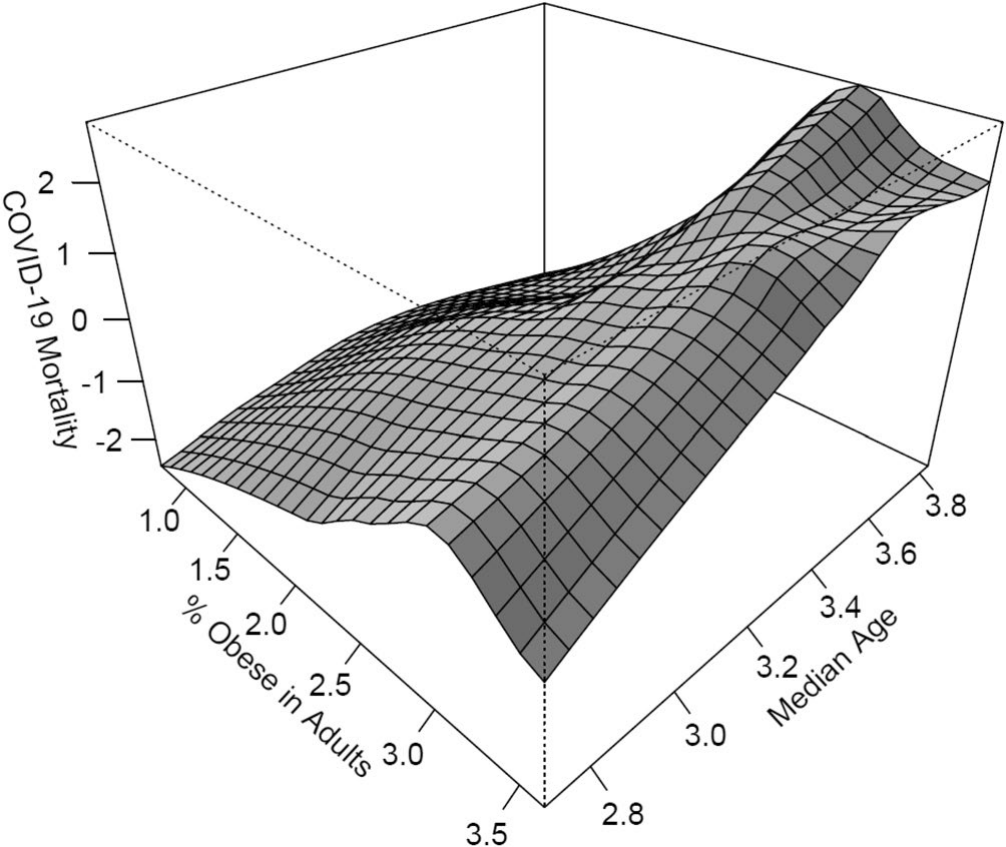
COVID-19 Mortality, Age, and Obesity.

We adhere to the clinical definition that an individual is obese if his or her BMI is at least 30 kg/m^2^ where the BMI is a person’s weight in kilograms (kg) divided by his or her height in meters (m) squared. At the outset, in keeping with the clinical knowledge that the more aging a population the higher its mortality since immune and inflammatory responses fall as an individual becomes older, we start by visualizing any association between COVID-19 mortality ($m$) and adult population proportion of obese ($p$) alongside the population’s median age ($a$). To do so, we estimate a non-parametric regression

$$\mathrm{log}m=f\left(\mathrm{log}p,\mathrm{log}a\right)+\varepsilon$$

for which Euclidean distances

$$E=\sqrt{\sum_{j=1}^{c}{\left({\mathrm{log}{\widehat{p}}_{j}-\mathrm{log}p}_{0}\right)}^{2}+\sum_{j=1}^{k}{\left({\mathrm{log}{\widehat{a}}_{j}-\mathrm{log}a}_{0}\right)}^{2}}$$

are used to define a multivariate neighborhood around a focal point $\left({\mathrm{log}p}_{0},\mathrm{log}{a}_{0}\right)$, where $\varepsilon$ is the error term; *c* is the number of observations (countries, listed in Appendix 1) indexed by *j*; and ($\mathrm{log}{\widehat{p}}_{j}$, $\mathrm{log}{\widehat{a}}_{j}$) denotes standardized regressor vectors. We run a weighted polynomial regression, the weights being $\left(\frac{E}{h}\right)$ where *h* is the half-width of the neighborhood. Repeating this procedure, for typical combinations of values of predictors, generates a regression surface. For each regressor, we take 50 values that are evenly spaced along the range of the variable, to create a data frame that consists of combinations of values of $\mathrm{log}p$ and $\mathrm{log}a$: the expand.grid function is used in R^11^. Next, we compute the corresponding fitted values on a regression surface and reshape the predicted values into a 50 by 50 matrix reflected on Fig. 4.

**Figure 5:**
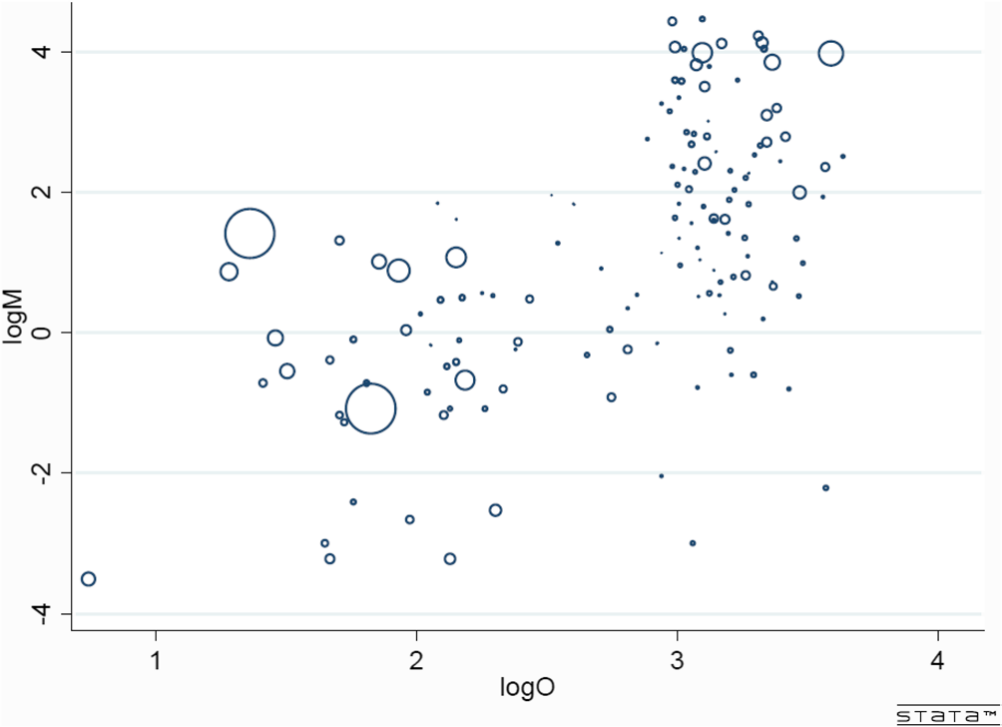
COVID-19 Mortality and Proportion of Obese in Adult Populations (142 countries hosting more than 7.5 billion people). Each circle, with radius proportional to population size, represents a country.

In view of the regression surface, it appears unlikely that variation in either of the regressors would have any significant effect on a partial regression in the direction of the other. The surface also hints at a positive association between the proportion of obese in a country’s adult population and mortality due to COVID-19. Figure 5 plots COVID-19 mortality against the percentage of obese in the adult population of each nation. The pattern emerging from this plot appears to be consistent with the regression surface.

In the following section, releasing any restrictions that non-parametric estimation may entail as a result of an expansion in the continuous variable space, we report our findings from a parametric regression

$$\mathrm{log}m={\beta }_{0}+{\beta }_{1}\mathrm{log}p++{\beta }_{2}\mathrm{log}a+\epsilon$$

where $\epsilon$ measures any error due to omitted explanatory variables.

## Results

We present convincing evidence of a significant positive partial correlation between the percentage of obese in adult populations and mortality due to COVID-19, after controlling for population characteristics, in Table 1.

**Table 1:** COVID-19 Mortality and Proportion of Obese in Adult Population.

Explanatory variable	Regression coefficient
log (percentage of obese in the adult population)	1.321***
log (median age)	1.319***
Constant	− 6.965***
Sample size	142
Adjusted R-squared	0.36
*F*-statistic	40.42

Dependent variable is log of COVID-19 Mortality.

***indicates that a regression coefficient is statistically significant at 1% level.

Tables 2 and 3 show that this association is stronger for high income countries, than it is for lower middle-income and low-income countries, while Table 4 shows that such an association is insignificant for upper middle-income countries. Bounds of confidence intervals, centered around the point elasticity estimates of elasticities of COVID-19 mortality with respect to the percentage of obese in adult populations, extend from 0.7 to 2.1 and is highest for the group of countries categorized as high-income: a rise in the obese adult population by 1 percentage point explains 1.5 percentage point rise in mortality due to COVID-19 for high income countries. The observed positive association between the percentage of obese in adult populations and mortality due to COVID-19 does not disappear in a multivariate framework to the extent that the sign and statistical significance of the partial correlation do not change, as demonstrated in Supplementary Table 1, when we alter the conditioning set of information by combining additional covariates.

**Table 2:** COVID-19 Mortality and Proportion of Obese in Adult Population: High-Income Countries.

Explanatory variable	Regression coefficient
log (percentage of obese in the adult population)	1.507***
log (median age)	2.006
Constant	− 9.987
Sample size	49
Adjusted R-squared	0.1
*F*-statistic	3.92

Dependent variable is log of COVID-19 Mortality.

***indicates that a regression coefficient is statistically significant at 1% level.

**Table 3:** COVID-19 Mortality and Proportion of Obese in Adult Population: Lower Middle-Income and Low-Income Countries.

Explanatory variable	Regression coefficient
log (percentage of obese in the adult population)	1.207***
log (median age)	1.354
Constant	− 6.874**
Sample size	54
Adjusted R-squared	0.26
*F*-statistic	10.29

Dependent variable is log of COVID-19 Mortality.

***indicates that a regression coefficient is statistically significant at 1% level and **indicates that a regression coefficient is statistically significant at 5% level.

**Table 4:** COVID-19 Mortality and Proportion of obese in Adult Population: Upper Middle-Income Countries.

Explanatory variable	Regression coefficient
log (percentage of obese in the adult population)	1.055
log (median age)	0.215
Constant	− 1.779
Sample size	39
Adjusted R-squared	0.01
*F*-statistic	1.22

Dependent variable is log of COVID-19 Mortality.

## Discussion

Our key contribution is founded not only on exploiting variations across a large sample of countries but lies in circumventing typical challenges faced by the extant literature that relies on individual level data as well. More specifically, inferences based on studies involving individual level data are subject to greater noise and lack uniformity in terms of design, methodology, sample, and/or patient treatment.

Clinically, an individual with a BMI ranging between 25 kg/m^2^ and 29.9 kg/m^2^ is considered *overweight* but not *obese* unless the BMI is at least 30 kg/m^2^. Our search for correlations between the percentage of the adult population that is *obese* and mortality due to COVID-19, is a natural follow up of Arulanandam et al.^12,13^ who reported a positive and significant correlation between the share of the *overweight* in adult populations and mortality due to COVID-19 spread across 154 nations.

**Figure 6:**
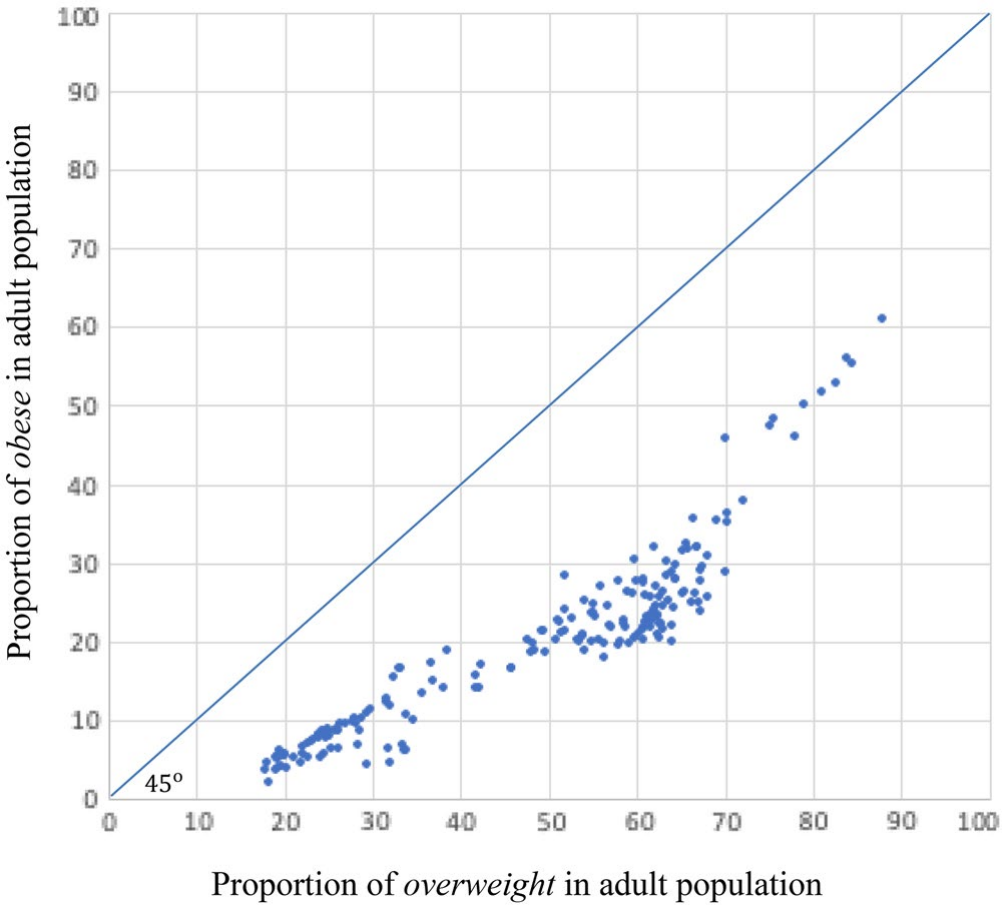
Proportion of Obese vs. Overweight in Adult Populations across Countries.

Our results are consistent with, albeit distinct from, those found in Arulanandam et al.^12,13^. We report a robust positive association between the percentage of the *obese* adult populations and mortality due to COVID-19, in this paper, while Arulanandam et al.^12,13^ report a robust positive association between the percentage of the *overweight* adult populations. Figure 6 plots the percentage of *obese* in the adult population of each nation against the percentage of *overweight* in its adult population.

Arulanandam et al.^12,13^ found that the bounds of confidence intervals, computed around the estimated point elasticities with respect to the percentage of the *overweight* in adult populations, extend from 0.2 to 5.4. We observe that the bounds of confidence intervals, computed around the estimated point elasticities with respect to the percentage of the *obese* in adult populations, are limited between 0.4 and 3.0: in comparison, the estimated point elasticities are more tightly distributed when computed with respect to the percentage of the *obese* in adult populations than they are when computed with respect to the percentage of the *overweight* in adult populations.

It would, of course, be remiss not to recognize that our study is subject to some typical caveats that stem from using cross-country regressions. Most importantly, any regression analysis presupposes that observations are drawn from a distinct population. As such, the validity of inferences drawn from any cross-country regression analysis can be questioned on the ground that countries may have very little in common that can merit including them in the same regression. Also, notwithstanding the credibility of the sources (listed in Appendix 2) that allow access to relevant data, inferences drawn in our study must be interpreted with caution since the quality and reliability of data on COVID-19 cases may be sensitive to reporting accuracies that can vary across countries.

## Conclusion

The timeliness of our contribution may be placed in the context of a quote from the *Scientific American* (2020), “We’re in a terrifying and confusing pandemic, with new and sometimes conflicting information about COVID-19 emerging all the time.” We hope to enrich interests in scientific inquiries, at the intersections of epidemiology and health economics, by reporting our findings on correlations between the percentage of obese in adult populations and mortality due to COVID-19 across nations. In particular, we observe a positive significant correlation between the percentage of obese in a nations’ adult populations and mortality due to COVID-19. The sign and statistical significance of this observed correlation holds across income groups and is not sensitive to population’s characteristics (e.g. age and/or gender distribution). The elasticity estimate of COVID-19 mortality, with respect to the percentage of obese in adult populations, is the highest among countries that belong to the high-income group. The bounds of confidence intervals extend from 0.7 to 2.1. A rise in the obese adult population by 1 percentage point explains 1.5 percentage point rise in mortality due to COVID-19 for high income countries.

## Supplementary Information

Supplementary Information.

Supplementary Table 1.

## Data Availability

The datasets used and/or analysed during the current study will be available from the corresponding author on reasonable request.
