# Supplementary material for: Obesity and COVID-19 mortality are correlated
Source: Sci Rep. 2023 Apr 11;13:5895. doi: 10.1038/s41598-023-33093-3 (PMC10088638; doi:10.1038/s41598-023-33093-3)
Supplement: Supplementary file 1 — Supplementary Information. [file 41598_2023_33093_MOESM1_ESM.pdf]

Appendices

Appendix 1. List of Countries in Sample

Afghanistan	Cote d'Ivoire	Iraq	Mozambique	Spain
Albania	Croatia	Ireland	Namibia	Sri Lanka
Algeria	Cuba	Israel	Nepal	Suriname
Angola	Cyprus	Italy	Netherlands	Sweden
Antigua and Barbuda	Czech Republic	Jamaica	New Zealand	Switzerland
Argentina	Denmark	Japan	Nicaragua	Tajikistan
Armenia	Djibouti	Jordan	Niger	Tanzania
Australia	Dominican Republic	Kazakhstan	Nigeria	Thailand
Austria	Ecuador	Kenya	Norway	Togo
Azerbaijan	El Salvador	Kuwait	Oman	Trinidad and Tobago
Bahrain	Equatorial Guinea	Latvia	Pakistan	Tunisia
Bangladesh	Estonia	Lebanon	Panama	Turkey
Barbados	Ethiopia	Lesotho	Papua New Guinea	Uganda
Belarus	Finland	Liberia	Paraguay	Ukraine
Belgium	France	Libya	Peru	United Arab Emirates
Belize	Gabon	Lithuania	Philippines	United Kingdom
Benin	Georgia	Luxembourg	Poland	United States
Bosnia and Herzegovina	Germany	Madagascar	Portugal	Uruguay
Botswana	Ghana	Malawi	Qatar	Uzbekistan
Brazil	Greece	Malaysia	Romania	Vietnam
Bulgaria	Guatemala	Maldives	Rwanda	Zambia
Burkina Faso	Guinea	Mali	Sao Tome and Principe	Zimbabwe
Cameroon	Guinea-Bissau	Malta	Saudi Arabia	
Canada	Guyana	Mauritania	Senegal	
Central African Republic	Haiti	Mauritius	Serbia	
Chad	Honduras	Mexico	Sierra Leone	
Chile	Hungary	Moldova	Singapore	
China	Iceland	Mongolia	Slovenia	
Colombia	India	Montenegro	Somalia	
Comoros	Indonesia	Morocco	South Africa	

Appendix 2. List of Variables used in Regression Analyses

Variable	Definition	Source
COVID-19 mortality	Deaths, by the end of 2020, from severe acute respiratory syndrome coronavirus 2 (COVID-19) per million people.	European Centre for Disease Prevention and Control
Percentage of obese adults in the population	Percentage of populations aged 18 and above, in 2020, with Body Mass Index of 30 kilograms per meters squared or more.	Global Health Observatory, World Health Organization
Income group dummy	Classification based on 2019 per capita Gross National Income (GNI): 1 = high income (per capita GNI of $12,536 or more); 2 = upper middle income (per capita GNI ranging from $4,046 to $12,535); 3 = Lower middle income (per capita GNI ranging from $1,036 to $4,045); and 4= Low income (per capita GNI of $1,035 or less).	World Bank
Median age	Median, expressed in years, based on the 2019 revision of age distributions.	World Population Prospects, United Nations
Percentage of elderly in the population	Percentage of population aged 65 and above, based on the 2019 revision of age distributions.	World Population Prospects, United Nations
Percentage of female in the population	Percentage of females in population, based on the 2019 revision of sex distributions	World Population Prospects, United Nations
