# Supplementary material for: Obesity and COVID-19 mortality are correlated
Source: Sci Rep. 2023 Apr 11;13:5895. doi: 10.1038/s41598-023-33093-3 (PMC10088638; doi:10.1038/s41598-023-33093-3)
Supplement: Supplementary file 2 — Supplementary Table 1. [file 41598_2023_33093_MOESM2_ESM.pdf]

Supplementary Table 1. COVID-19 Mortality and Proportion of the Obese in Adult Population: Robustness

Regression	1	2	3	4	5	6	7	8	9
log (percentage of obese in the adult population)	1.697***	1.232***	1.222***	1.238***	1.233***	1.235***	1.220***	1.233***	1.262***
Income group dummy (1=high income; 2=upper middle income; 3=lower middle income; 4=low income)		-0.424***	-0.220	-0.270	-0.426***	-0.250	-0.203	-0.082	-0.135
log (median age)			0.822			0.170	0.872		-1.997
log (percentage of elderly in the population)				0.293		0.250		0.586*	1.368*
log (percentage of female in the population)					0.149		-0.332	-2.475	-4.877*
Constant	-3.595***	-1.386	-4.550	-2.316**	-1.966	-2.837	-3.456	6.376	20.919
Sample size	142	142	142	142	142	142	142	142	142
Adjusted R-squared	0.325	0.356	0.358	0.360	0.352	0.356	0.354	0.363	0.388
F-statistic	68.95	40.03	27.23	27.47	26.50	20.46	20.30	21.12	17.21

Notes: Dependent variable is log of COVID-19 Mortality.

*** indicates that a regression coefficient is statistically significant at 1% level

** indicates that a regression coefficient is statistically significant at 5% level; and

* indicates that a regression coefficient is statistically significant at 10% level.
